# Kinetic instability, symmetry breaking and role of geometric constraints on the upper bounds of disorder in two dimensional packings

**DOI:** 10.1038/srep26968

**Published:** 2016-06-01

**Authors:** Raj Kishore, Shreeja Das, Zohar Nussinov, Kisor K. Sahu

**Affiliations:** 1 School of Minerals, Metallurgical and Materials Engineering, Indian Institute of Technology, Bhubaneswar-751007, India; 2 Department of Physics, Washington University in Saint Louis, MO-63130-4899, USA

## Abstract

Although the energetics of grain boundaries are more or less understood, their mechanical description remains challenging primarily because of very fast dynamics in the atomic length scale. By contrast, granular dynamics are extraordinarily sluggish. In this study, two dimensional centripetal packings of macroscopic granular particles are employed to investigate the role of geometric aspects of grain boundary formation. Using a novel sampling scheme, the extensive configuration space is well represented by a few prominent structures. Our results suggest that cohesive effects “iron out” any disorder present and enforce a transition towards a “fixed point” basin associated with a universal high density jammed hexagonal structure. Two main conjectures are advanced: (i) the appearance of grain boundary like structures is the manifestation of the kinetic instabilities of the densification process and has its origin in the structural rearrangement and (ii) the departure from six-fold coordination in the final packing is bounded from above by a sixth of the angular dispersion present in the initial configuration. If similar predictive consequences are further developed for three dimensional cases, this may have far reaching consequences in many areas of science and technology.

Grain boundaries are fascinating entities that may significantly alter material properties. While, typically, their influence on the electrical^1^,^2^,^3^ and thermal properties^4^ is moderate, the mechanical^5^,^6^ and optical^7^,^8^ properties may be spectacularly impacted by grain boundaries. Although the energetics (thermodynamic aspects) of grain boundary are more or less well understood^9^, their mechanical description is still lacking. The very rapid atomic scale dynamics during solidification from the liquid state^10^ renders many such questions not too crucial. Glassy dynamics, on the other hand, are orders of magnitude slower as these occur in a (semi-)rigid state^11^,^12^. Glassy dynamics are notoriously difficult as they include many complicating factors, whose effects are poorly understood^13^,^14^. In this study, centripetal packing is used to investigate the role of geometric constraints in the mechanical description of grain boundary formation, particularly the initial stages, because of its slow dynamics. Slow dynamics primarily arise due to the low energy densities of granular particle assemblies as compared to their atomic counterparts (roughly five orders of magnitude smaller). Additionally, the potential energy landscape (PEL) in atomic systems possess relatively deep minima near the crystalline (ordered) configurations^15^,^16^. Therefore, in the configuration space, if a system is in the neighbourhood of any such ordered state, the system inevitably veers towards it. These minima act as local attractors in PEL and it is very difficult to study dynamics in this space without falling into these steep and deep wells. The main objective of this work is to study the effects of geometric constraints, particularly those imposed by the linear dimensions of the finite atoms, on the mechanical description of grain boundary formation. In order to eliminate complexities arising from pair-wise interactions of long range potentials, cohesion is modelled via an externally applied simple centripetal force field. This is particularly advantageous as the absence of pairwise interactions moderates the depth of these wells in the PEL; proximate systems therefore can be readily studied. Monoatomic systems are ideal starting point as they involve fewer geometrical complexities.

The present study relates to a monodisperse sphere packing problem^17^,^18^, which is an interesting science in its own right. Sphere packing is presumably one of the oldest problems mankind has ever attempted to study dating back to pre-historic times because of its implication in measurement of food grains by pouring them in a basket^19^. A precise and complete understanding of such an apparently simple problem (the structure of the heap) is still out of reach for modern science. However, considerable simplicity can be achieved by imposing the constraint that all the constituent particles consist of monodispersed spheres alone. With the advent of X-ray crystallography, the intricate details of atomic packing in crystals could be obtained and those atomic ensembles were almost always modeled using sphere packing. The aperiodic arrangement of sphere packing also gained importance as it was shown that hand-made ball-and-stick models of monodisperse spheres have significant structural similarity with the atomic structure of metallic (pure) liquids as studied by X-ray diffraction^20^,^21^,^22^. Mathematical treatment for mono-disperse sphere ensembles, on the other hand, faced considerable challenge both for periodic and aperiodic arrangements. “While all the physicist and engineers knew” that using mono-dispersed spheres, a denser packing than hexagonal close packed or face-centered cubic systems, both having 74% packing density, could not be achieved for large volumes. Proving this mathematically turned out to be a formidable challenge^23^. So much so, that it featured in the list of greatest unsolved problems in mathematics^24^. Only in 1998, a mathematical proof to this conjecture was presented^25^. Another important development has taken place in the theoretical formulation of jamming^26^,^27^,^28^,^29^,^30^,^31^. Other than those limits, generalized predictions without resorting to detailed numerical experiments about sphere systems have largely remained elusive.

In the present study, the overall symmetry of disparate systems will be dictated by the initial structure because of rotational invariance of the centripetal force field. It is observed that, packing is achieved by breaking this symmetry and making a transition to a denser topological class which is hexagonal for 2-D disks. A kinetic instability underlies this phenomenon and leads to symmetry breaking. New (dis)order parameters also enable predictions for final structures without resorting to detailed experiments/simulations, which have thus far eluded sphere-packing studies. In addition, this article aims to obtain fundamental insights about the role of geometric constraints on the mechanism of grain boundary formation in 2D, using simple systems consisting of monodispersed disks where, cohesion is not because of complex pairwise interactions, but because of a global hypothetical force field acting toward the center of the system. Real materials are three dimensional and hence findings of this study will not have immediate applicability. It, however, attempts to chart a path towards that goal. If such predictive capabilities are further developed for the three dimensional cases, it will have far reaching consequences to earthquake and avalanche studies^32^,^33^ and to industries such as pharmaceutical^34^,^35^, minerals^36^, agricultural^37^, cement and concrete^38^, chemicals^39^ etc.

## The Model System

Each simulation involves roughly 10000 mono-dispersed particles distributed in a 2-D box with low initial density (~12% packing fraction). At time *t* = *0*, all of the particles are subjected to an externally applied centripetal force (magnitude set equal to gravitational force) directed towards the center of the box. The particles lose energy during collisions among themselves owing to the friction and damping, which are typical for realistic granular particles. Finally a packing is obtained. The present study aims to capture the effect of initial state sampled anywhere from the entire configuration space that encompasses both periodic as well as aperiodic regimes having similar (~12%) packing density. It is established that for 2-D monodispersed ordered states, only five types of Bravais nets (namely square, hexagon, rectangle, centred rectangle and oblique) belonging to four crystal systems (namely square, hexagon, rectangle and oblique) do exist^40^. The present study involves all five Bravais nets as well as random structure as initial configurations. More details concerning the uniqueness of random configuration are provided in the Supplementary S1.

We will study the effect of the perturbation of initial particle positions relative to an initial order (i.e., positional disorder). In two dimensions, there are only two crystal classes, namely ‘*compact-hexagonal’* (defined below) and ‘*compact-square*’ lattices having a unique length scale (both basis vectors have same length). The word ‘*compact*’ in this article implies regularly packed structures, where neighboring particles touch each other (unlike the low density configurations prior to inward collapse in present study wherein neighbors are widely separated). However, the first neighbor distances between the lattice points for both these *compact* crystals are also identical. The perturbation is quantified by the difference in the distance between second neighboring particles in those two lattices. The difference in the second neighboring distances in *compact* hexagon and square structures is 2(√3−√2)*r* = 0.6537*r* and this value will be referred to as a perturbation of size 100%. Four different perturbation levels were used in the present work: none, 50%, 100% and 150%. Though the perturbations used in present study in this scale are high, they are roughly one order of magnitude smaller than the initial inter-particle separation. Because of this, after adding perturbations the derived structures are assumed to belong to the original class for easy classification purpose (nomenclature) though these structures no longer possess the original symmetries in a strict mathematical sense.

One might suspect that the PEL of 2D atomic ensembles might contain deep wells near the five Bravais nets and hence transitions from one to other might be energetically costly. The absence of interatomic force in present simulations is a clear distinction from the atomic analogues; the motivation for avoiding such local trapping led to the choice of *uniform* perturbation (meaning all particles are displaced by identical value decided by perturbation level but in random directions). To generate sufficient statistics, for each symmetry type and perturbation levels, five simulations were performed, which were realized by random assignment of perturbation directions. Generating other instances of simulation is trivial for perturbed systems because of random assignment of perturbation directions for individual particles. For unperturbed systems, lack of such movement and the requirement of fixed initial packing density is an issue as it does not allow any change of inter-particle distance for ordered structures. Also, for these cases, one cannot expect to generate other instances of simulation by rotating an original configuration because of rotational invariance of central force field. The central nature of the force, however, destroys the translational invariance and thus distinct instances of initial configurations for unperturbed systems were generated by translating the original configurations by small values (~0.1*r*) [see Supplementary S2].

**Figure 1:**
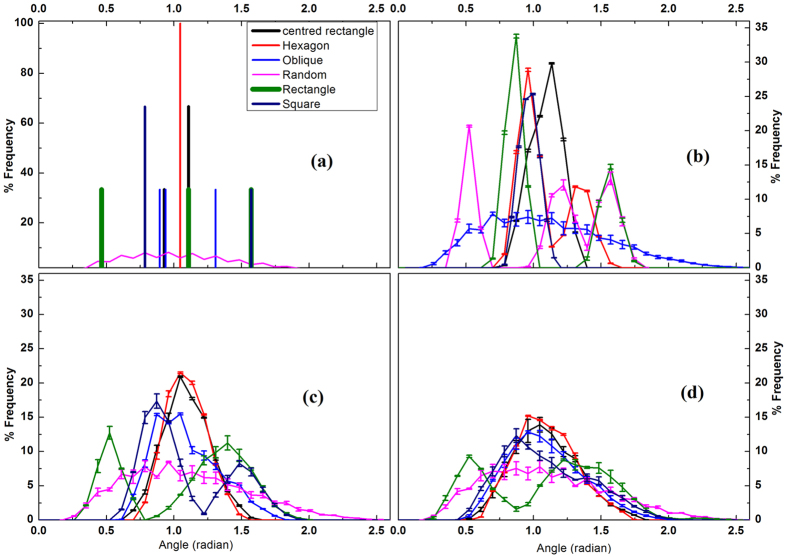
Angular distribution of Delaunay triangles for different initial configurations: (**a**) 0% perturbation (**b**) 50% perturbation (**c**) 100% perturbation (**d**) 150% perturbation applied to various reference states (centered rectangle, …, square lattice). For high perturbations, the angular distributions for different lattice-classes become similar. This property underlies the basis of an interpolation scheme between different lattice-classes and random configurations (See text).

Since the initial densities and hence the average inter-particle distances are kept constant during all the simulations, one of the key parameters of the initial configuration that is expected to influence the final packed structure is the distribution of angles between “neighboring particles”. An objective definition of “neighborhood” is provided by a Voronoi diagram. A Voronoi diagram centered about sphere *i* encloses all points *P* that are closer to it than any other sphere *j*. Spheres *i* and *j* whose respective Voronoi regions share a face are termed “natural neighbors”. Delaunay triangulation^41^ produces a graph of connected natural neighbors by constructing the geometrical dual of the Voronoi tessellation. Therefore, the distribution of angles of a Delaunay net is one of the best representation schemes for the three body distribution function and will be used in the present article. An advantage of this approach is the insensitivity to scaling (the results are only function of angular distribution and are independent to the inter-particle distances) making the method applicable for dilute (gas-like) and dense (solid-like) phases and even for a mixture of both, which is difficult to analyze by other methods. The distribution of angles of Delaunay nets is shown in Fig. 1. At higher perturbation levels, the differences in angular distributions between Bravais nets and random configurations get effectively blurred ensuring a smooth transition from crystal geometries to random configurations. The sampling scheme presented here using different levels of perturbation helps interpolate between lattice types and enables probing the entire configuration space for low initial system density using only 21 configurations [see Supplementary S3] though more than 120 configurations were studied for statistical considerations. This methodology of using perturbation as an interpolation scheme between Bravais lattices can be easily extended to three dimensions for representing the extensive configuration space (particularly for low density regimes) using limited numbers of samples. Since an arbitrary displacement of any particle results in a different configuration, the configuration space itself is *‘uncountably infinite’* and the present scheme allows to completely sample the entire configuration space (for the invariant density) by as low as few tens of discrete structures.

## Methods

Distinct Element Modeling (DEM) is a method of choice for realistic simulation of ‘soft’ particles and has been implemented in the standard form^42^,^43^,^44^,^45^,^46^ for the present study. Here, particle motions are calculated by numerical integration of the Newtonian equations of motion. Normal component of pairwise contact dynamics is modeled by Hertz method modified for viscoelastic spheres^47^,^48^ and tangential component is based on Haff and Werner^49^ model. A time step of 1 *μ*sec is used for the present simulation. Contact detection is performed using a Verlet list^50^,^51^. Numerical integrations are performed by the fifth order predictor corrector method^52^. The details of mathematical formulations and numerical schemes are discussed in details in Supplementary S4.

## Results

### Dynamical analysis of packing

**Figure 2:**
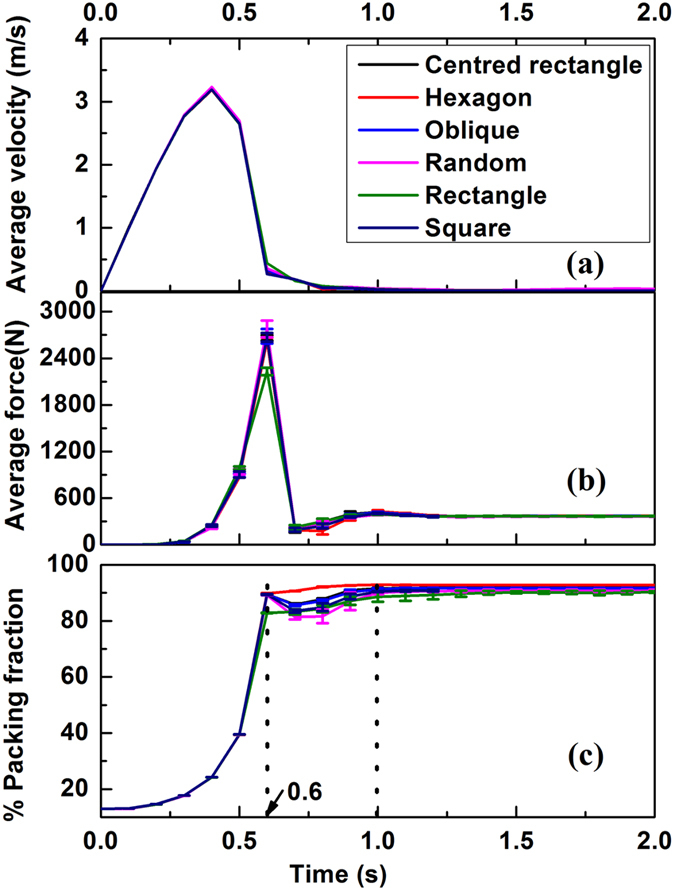
Temporal evolution for each symmetry class in the absence of a perturbation. (**a**) Average velocity and (**b**) force on particles (**c**) the density (measured via Delaunay nets) as a function of time. Two vertical lines in 0.6 s and 1.0 s indicate the time span where the evolution of density is different and is indicative of varying degrees of structural rearrangements. The trajectories of all the particles for two systems exhibiting maximum differences, namely hexagonal and random systems are plotted in Fig. 3. The error bars mark the standard deviations.

**Figure 3:**
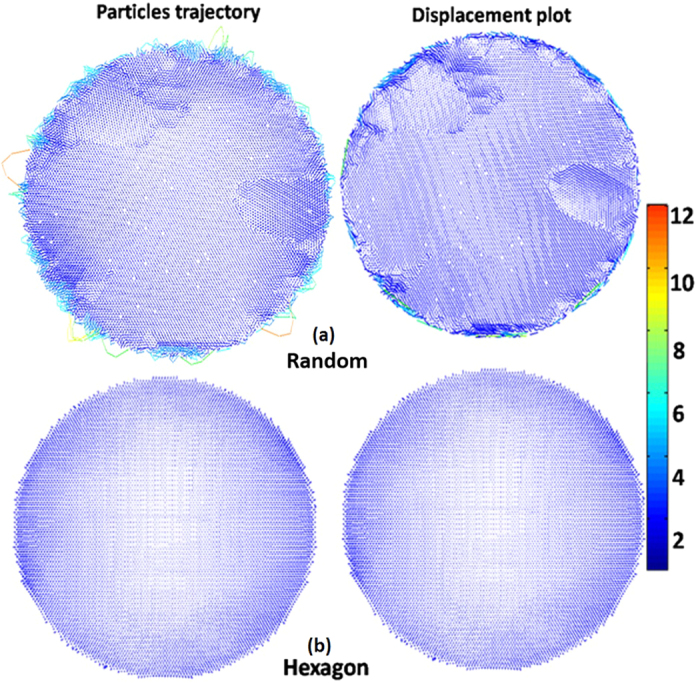
Trajectory of particles between 0.6 s and 1.0 s for (**a**) random and (**b**) hexagonal systems. The trajectories are updated each 0.05 s. Bar represents color coding used for the length of both trajectories and displacements in particle diameter unit for each particle during the time interval of 0.6 to 1 sec.

Different aspects of evolution of the configurations with no perturbation are presented in Fig. 2(a–c). While the average velocity and force (Fig. 2a,b) display nearly identical patterns (with the exception of rectangle in Fig. 2b); in the interval between 0.6 to 1 second, the density (Fig. 2c) exhibits different trends. This disparity is indicative of varying structural rearrangements. The results reflect the fact that kinetic evolution of the systems is independent of the symmetry of the original state apart for weak signatures of structural rearrangements. For a better understanding of the origin of structural rearrangements, the trajectories of all the particles for two configurations that exhibit the largest discrepancy (namely those systems with initial hexagonal and random structures) are depicted in Fig. 3a,b respectively. The random system is more mobile as compared to the hexagonal system (Fig. 3a,b respectively). A particularly striking feature is the presence of domain like structure for the system with a random initial configuration and their absence in the hexagonal case. Further implications of such structures will be discussed later.

### Statistical analysis of final packing (Statics)

**Figure 4:**
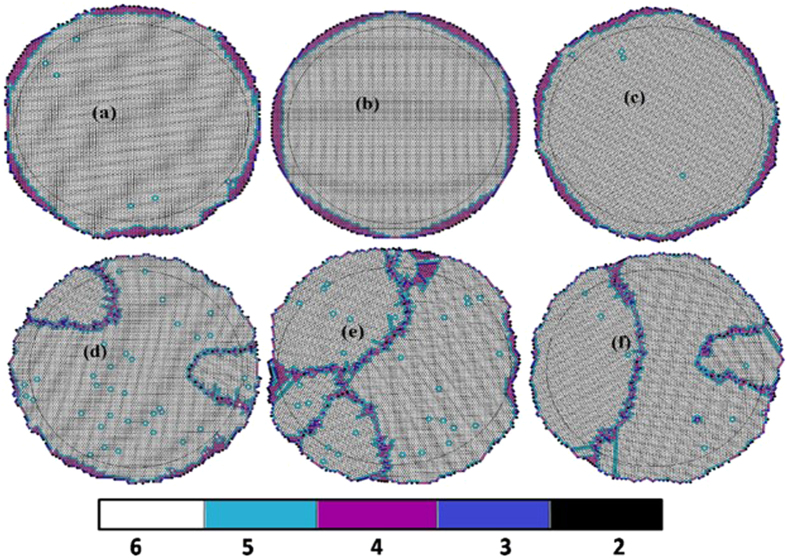
Coordination number distribution in final packed structure with initial configuration as (**a**) Centered rectangle (**b**) Hexagon (**c**) Oblique (**d**) Random (**e**) Rectangle (**f**) Square. Color bar shows particles with different coordination numbers.

The final packed structures for six different initial configurations (one set) are shown in Fig. 4. Irrespective of the symmetry of initial configurations, the final packings for all cases are predominately hexagonal with minor but varying degrees of departures from six fold coordination. Such departures are of the form of point and line defects. The line defects are reminiscent of grain boundary like structures one typically finds in metallic microstructures (ceramic/polymer microstructures are rather complex compared to the atomic description of grain boundaries in pure elemental metals). It is observed that the packing obtained from initial hexagonal configurations have the least disordered regions. It is interesting to compare the trajectories depicted in Fig. 3 with the final configurations of random and hexagonal packings shown in Fig. 4. One can immediately establish the connection between the domain boundaries in Fig. 3a and the grain boundary like structure for random configuration in Fig. 4d (and the corresponding absence of similar features for hexagon in the Figs 3b and 4b). Therefore, it can be concluded that line defects corresponding to departures from six fold coordination, manifest as grain boundary-like structures, are generally correlated to differing degrees of structural rearrangements and have a kinetic origin. This establishes a connection between kinetic evolution and final structure.

The evolution of the rotational symmetries during the course of simulation is of particular interest. Each simulation in the present study captures three distinct aspects: (i) the central force, which is rotationally invariant, (ii) the initial structures which show limited rotational symmetries as dictated by the corresponding Bravais nets (for random, there is none) and, (iii) the governing laws (e.g., Newton’s laws, collisions, etc.), which are rotationally invariant. An entire simulation for a particular configuration is thus limited by the symmetries of the initial configuration. Hence the appearance of line defects that breaks the rotational symmetries even for Bravais nets deserves serious attention [see Supplementary S5]. Grain boundary like structures reflect the kinetic instabilities of the densification process that originate in the present case by symmetry breaking during structural rearrangement process.

A detailed analysis of the final packings appears in Supplementary S6. In a 2-D densification process, there is a natural tendency for the particles to achieve a six-fold coordination as such coordination can maximize the density. The externally applied centripetal force emulates a cohesive force driving the system towards maximum density. Because of this, irrespective of the initial configuration, most of the particles in final packing tend to have a six-fold coordination. However, a six-fold coordination corresponds to a crystalline order (Hexagonal). Hence we introduce two new (dis)order parameters namely (i) the *Bernoulli Entropy* (*H*) (equation (1)) and (ii) the *degree of disorder* (*D*) (equation (2)). These two measures monitor the departure from the six fold coordination. The *Bernoulli Entropy* (*H*), which is the Shannon entropy^53^,^54^ for the simple case of binomial distribution is associated with the following binary process: whether a particle deviates from six fold coordination or not. The *Degree of disorder* (*D*) quantifies how large the departure is (including its magnitude). Both of these parameters are normalized which makes them independent of sample size.


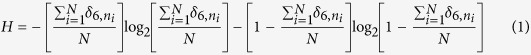



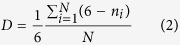


**Figure 5:**
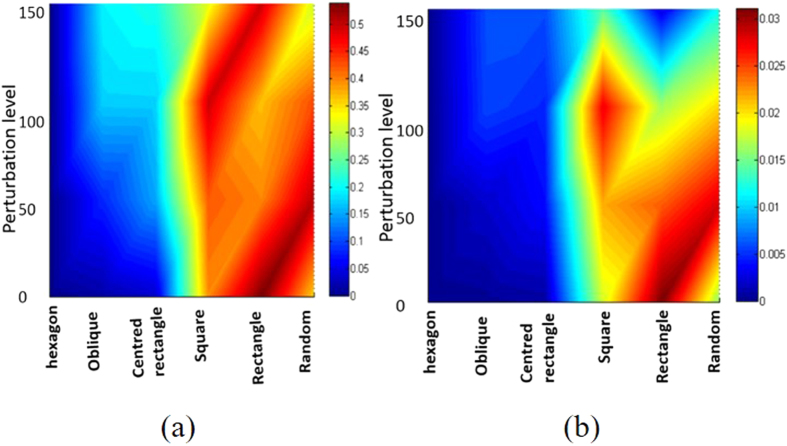
(**a**) Entropy and (**b**) disorder (both color coded, and averaged over five sets) as a function of both symmetry of initial configuration and perturbation level. The data points are only at grid values (averages) and the intervening spaces are color-codded with smooth interpolating spline for a guide to the eye to identify patterns.

Here, *n*_*i*_ is the coordination number of *i*^*th*^ particle and *N* is the total number of particles in the configuration and *δ* is Kronecker delta. The complimentary functions *H* and *D* can assume any value between zero and unity and are somewhat correlated (see Supplementary S7 for details). Figure 5a,b shows both *H* and *D* as a function of symmetry class and perturbation strengths.

We earlier argued that an angular dispersion might play a significant role and this will be closely examined now. The angular dispersion can capture the evolution of disorder over the entire dynamic range. In contrast, the entropy *H* and the degree of disorder, *D*, assume meaningful values at high densities when most particles are in contact. The choice of the triangular Delaunay nets as a basis for the measurements of angles stipulates the mean value to be trivially π/3. A better characterization parameter of the distribution of the angles will then be given by the second moment (standard deviation) which will be referred here as angular dispersion and measured in radians.

**Figure 6:**
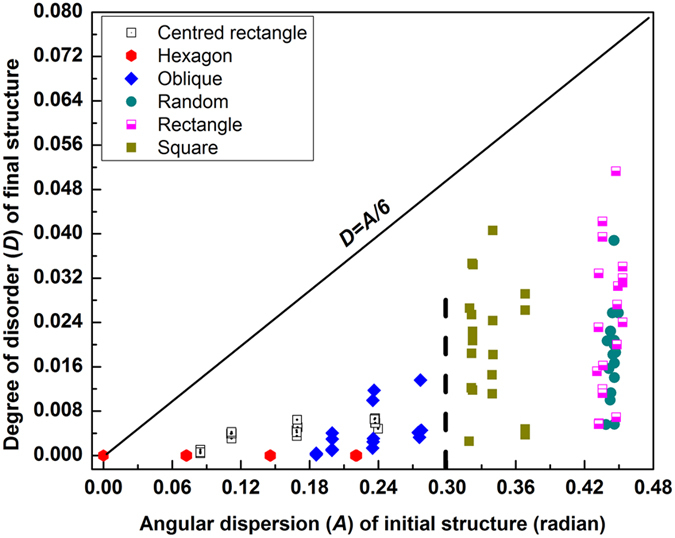
For all the simulations, the degree of disorder, *D* of final structures is plotted as a function of angular dispersion (measured in radians) of initial configurations. It is observed that degree of disorder, *D* of final structures are always less than one sixth of angular dispersion of initial configurations. Lower disorder in the final configurations that were obtained from hexagonal, oblique and centred rectangular structures (Fig. 5) can be explained by differences in their initial angular dispersion highlighted by a verticle dashed line.

Figure 6 demonstrates that the degree of disorder in final packings is always smaller than one sixth of the angular dispersion of initial states (for all possible symmetries). Albeit similarities, differences remain between the disparate initial states. This tendency towards a reduced degree of disorder is expected as the system becomes more compact. The effect of the centripetal force is to increase the density. The resultant dynamics not only “iron out” the disorder in the initial state but also enforce a topological transition towards six-fold hexagonal structure. In the high density limit, the system may progressively veer towards a “fixed point” basin associated with universal high density jammed structure. Nevertheless, correlations between the initial and final state do persist as the Fig. 6 elucidates (such as point and/or line defects seen in Figs 3 and 4).

In the middle of last century, Turnbull’s^55^ demonstration that liquid metals can be considerably super-cooled was surprising given their similarity in following aspects: (i) density (ii) coordination numbers of the liquid and crystalline phases (iii) their simple symmetric structural units, in contrast to the complex molecular structures, say, in organic compounds, proteins, etc. A recent study^56^ demonstrated that considerable structural similarity exists between the atomic ensemble of pure super-cooled liquid metals and the packing of macroscopic spheres. This result is striking not only because of the fact that the length scales involved differ by nearly eight orders of magnitude, but also because of the stark contrast in the nature of interaction between the constituent particles. While the atoms possess long range interactions, granular particle experience forces only when they are in contact. It highlights that the topological aspects (invoking the atom-bonding network) of sphere packing is of paramount interest and is of no lesser significance compared to interaction mechanisms/potentials. If and when present, the character of correlations and absence of very strong correlations between the dynamics and initial structure may extend beyond the centripetal systems investigated here. For instance, different classes of initial favored structures with varying degrees of commensurability were recently seen to lead to long time dynamics^57^. Hence, in the perspective of other works, there are strong indicators (that still need to be rigorously tested) hinting at a unifying picture for all polycrystalline materials.

The present observation provides predictive power for compaction of 2-d mono-sized spheres [prediction of disorder in final structure is discussed in details in Supplementary S7]. To the best of our knowledge, this is the first study explicitly illustrates how disorder of the final structure can be predicted from the initial structure. We now advance a second hypothesis for all 2d mono-sized spheres (disk) systems, irrespective of their type of structure that have low initial densities. Our results suggest that under the influence of long range forces in these systems, the departure from six fold coordination (i.e., the degree of disorder as defined in equation (2)) in final packing is always less than one sixth of the angular dispersion (when the latter is measured in radians) of the initial configuration as quantified by Delaunay triangulation. The lower disorder present in the final configurations obtained from hexagonal, oblique and centred rectangular compared to the square, rectangular and random counterparts (Fig. 5) can be explained by differences in their initial angular dispersion highlighted by a verticle dashed line in Fig. 6.

## Conclusions

2-D containerless packing of monodisperse spheres having very low initial density (~12%) have been studied. Using a novel sampling scheme, the nearly infinite 2-D configuration space for low initial packing fraction has been well represented and reasonably sampled by only 21 structures, though more than 120 simulations are performed for statistical considerations. This methodology can be used for interpolation between lattice and amorphous configurations and can be extended to three dimensions. In this article, densification process under the influence of centripetal force (set equal in strength to the gravitational force) is studied by the discrete element method. This external force serves as a tool to investigate the role of geometric aspects in the mechanism of grain boundary formation, particularly its initial stages because of its slow dynamics. All of the simulations show similar kinetic evolution, which, not only “irons out” the disorder in the initial state but also enforces a topological transition towards six-fold hexagonal structure leaving a weak signature of structural rearrangements. Despite this ‘ironing-out’, signature of initial structures persists in final structure in weak and disguised form. In the high density limit, the system progressively veered towards a “fixed point” basin associated with universal high density jammed structure. Analysis of the symmetry breaking led to the following conjecture: the appearance of grain boundary like structures reflects the kinetic instabilities of the densification process and has its origin in the structural rearrangements. An analysis of the structural disorder in final packing lead to another conjecture: the departure from six fold coordination per particle (degree of disorder as defined in equation (2)) in final packings is always less than one sixth of the angular dispersion (as measured in radians) of the initial configuration measured using Delaunay triangulation. This enabled predictions of disorder in final structures without resorting to detailed experiments/simulations, which has thus far eluded the packing studies.

## Additional Information

**How to cite this article**: Kishore, R. *et al.* Kinetic instability, symmetry breaking and role of geometric constraints on the upper bounds of disorder in two dimensional packings. *Sci. Rep.* **6**, 26968; doi: 10.1038/srep26968 (2016).

## Supplementary Material

Supplementary Information
