# Supplementary material for: Kinetic instability, symmetry breaking and role of geometric constraints on the upper bounds of disorder in two dimensional packings
Source: Sci Rep. 2016 Jun 1;6:26968. doi: 10.1038/srep26968 (PMC4887881; doi:10.1038/srep26968)
Supplement: Supplementary Information [file srep26968-s1.pdf]

Supplementary information

Kinetic instability, symmetry breaking and role of geometric constraints on the upper bounds of disorder in two dimensional packings

Raj Kishore[1], Shreeja Das[1], Zohar Nussinov[2], Kisor K. Sahu[1*]

1. School of Minerals, Metallurgical and Materials Engineering, Indian Institute of Technology, Bhubaneswar-751007, India
2. Department of Physics, Washington University in Saint Louis, MO-63130-4899, USA

*Correspondence to *kisorsahu@iitbbs.ac.in*

S1: The issue about uniqueness of random configuration

One might argue that there is no unique configuration that can be identified as random. The low initial packing density used in present study helps to get around this issue using following logic: the large neighbouring distance compared to particle radius (the ratio is 5.6*r* in the present case, and hence large mean free path) ensures that the packing retains its random character and can be used as a representative one. This will be further consolidated by the use of 'additional perturbation' in the initial configuration that will be discussed in the following section.

S2: Generating initial configurations for statistical purposes

To generate enough statistics, for each type, five simulations were performed. Because of the use of central force, the systems are rotationally invariant and one cannot expect to generate another instance of simulation by rotating an original configuration by an arbitrary amount. The central nature of the force, however, destroys the translational invariance and distinct instances of initial configurations were generated by translating the original configurations by small values (~0.1*r*). While generating five sets of initial configuration is a trivial job for perturbed systems, it is a tricky job for the unperturbed systems as the density and hence the lattice parameters of the initial configurations were kept constant. The original unperturbed Bravais nets (henceforth will be called master nets) were translated by very small amounts in different direction ([$X=X+0.1r$, Y];[X,$Y=Y+0.1r$]; [X=$X+0.1r$, $Y=Y+0.1r$]; [$X=X+0.05r$, $Y=Y+0.05r$];where, $X\in[xi, yi]$ $\forall i$, $(xi, yi)\equiv$ centre of ith particle) to generate different initial configurations without changing lattice parameters. This is again trivial for the random case. One can take a note that the translation vectors are order of magnitude smaller than the

physical dimension of the system, particles and even compared to the initial average inter particular distances. The perturbed systems were generated exclusively from the master nets only.

S3: Sampling the entire low density configuration space

So in total, six different topological classes (five Bravais nets and one random) and each with four different perturbation levels (none, 50%, 100% and 150%) and for each levels five instances (for generating statistics) were selected as initial configurations, so in total 120(=6×4×5) simulations were used for the present study. Using this novel sampling scheme, the nearly infinite 2-D configuration space has been well represented and reasonably sampled by only 21 (=6×4-3; as the random structure with three perturbation levels are still random and hence redundant) structures if one does not care for statistics.

S4: Methods in details

Packing algorithm

Distinct Element Modeling (DEM) is a method of choice for realistic simulation of 'soft' particles and has been implemented for the present study. The DEM scheme that is implemented in the present work is standard one[1-6]. The details of mathematical formulations and numerical schemes are still discussed in this section for the sake of completeness. Here particles motions are calculated by numerical integration of the Newtonian equations of motion.

The particle movement is composed of translational motion and rotational motion and are given by equations (S4.1) and (S4.2) respectively.

$$\frac{d\vec{v}}{dt} = \vec{F}/m \qquad \text{(S4.1)}$$

$$\frac{d\vec{\omega}}{dt} = \vec{T}/I \qquad \text{(S4.2)}$$

Where, $\vec{v}$ is the particle velocity vector, $\vec{F}$ is the total force acting on the particle, m is the mass of the particle, $\vec{\omega}$ is the angular velocity vector, $\vec{T}$ is the net torque developed due to contact forces and I is the moment of inertia of the particle. The total force acting on particle is the sum of external applied centripetal force and the contact forces due to interaction of

particle with the other particles of the system. Contact forces are normal and tangential forces.

Modelling of contact forces

In soft particle DEM model, where non-zero overlap (ξ_{ij}, equation (S4.3)) is allowed, contact force is a function of relative displacement or overlap between the particles when in contact.

$$\xi_{ij} = R_i + R_j - \left|\vec{r}_i - \vec{r}_j\right| \qquad \text{(S4.3)}$$

Where, R_i, R_j is the radius and $\vec{r}_i$, $\vec{r}_j$ is the position vector of the two interacting particles. The normal component of the contact force is repulsive in nature and its magnitude is directly proportional to the magnitude of the mutual compression. Hertz model[7] is modified for viscoelastic spheres[8] and used for calculation of normal force component of elastic spheres which was further generalised for viscoelastic spheres by including the damping factor along with elastic factor and is given by the equation (S4.4).

$$F_{el}{}^{n} = \max\left\{0, \left[\frac{2\mathrm{Y}\sqrt{R^{eff}}}{3\left(1-\upsilon^2\right)}\left(\xi^{3/2} + A\sqrt{\xi}\,\frac{d\xi}{dt}\right)\right]\right\} \qquad \text{(S4.4)}$$

The tangential force component has been calculated based on the model given by Haff and Werner[9]. Coulomb friction law is embedded in this model.

$$F^{t} = -sign\left(v_{rel}{}^{t}\right)\cdot \min\left(\gamma^{t}\left|v_{rel}{}^{t}\right|, \mu\left|F^{n}\right|\right) \qquad \text{(S4.5)}$$

Where, γ^t is tangential damping constant and μ is the coefficient of friction.

Numerical scheme

At the start of the simulation, particle position, velocity, and other higher order derivative of position is initialised (time t =0). Timestep of 1 μsec is used for the present simulation. In the time increment loop, first the prediction of all the parameters are done using Taylor series expansion. Since collision detection is time consuming, it is done through Verlet list[10,11] for all the particles. After collision detection is performed, total force on each particle is calculated. If a particle is not interacting with any other particle than the total force on the particle in question is only the central force otherwise total force is the resultant of central force and interaction forces (normal and tangential). Next, linear $\ddot{\vec{r}}^{\,corr}$ and angular accelerations are calculated by using Newton's law of motion (equations (S4.1) and (S4.2)).

Generally the values of these accelerations differ from predicted quantities. The difference in the two acceleration values (equation (S4.6)) is the measure for the deviation of the predicted coordinates, predicted velocity and other higher order derivatives from their true values. Correction of position, velocity and other higher order derivatives are done by adding numbers which are proportional to the deviation (equation (S4.7))

$$\Delta\ddot{\vec{r}} = \ddot{\vec{r}}_i^{corr} - \ddot{\vec{r}}_i^{pr} \qquad \text{(S4.6)}$$

$$\begin{pmatrix} \vec{r}_i^{corr}(t+\Delta t) \\ \vec{V}_i^{corr}(t+\Delta t) \\ \ddot{\vec{r}}_i^{corr}(t+\Delta t) \\ \dddot{\vec{r}}_i^{corr}(t+\Delta t) \\ \bullet \\ \bullet \\ \bullet \\ \bullet \end{pmatrix} = \begin{pmatrix} \vec{r}_i^{pr}(t+\Delta t) \\ \vec{V}_i^{pr}(t+\Delta t) \\ \ddot{\vec{r}}_i^{pr}(t+\Delta t) \\ \dddot{\vec{r}}_i^{pr}(t+\Delta t) \\ \bullet \\ \bullet \\ \bullet \\ \bullet \end{pmatrix} + \begin{pmatrix} C_0 \\ C_1 \dfrac{1}{\Delta t} \\ C_2 \dfrac{2}{\Delta t^2} \\ C_3 \dfrac{6}{\Delta t^3} \\ \bullet \\ \bullet \\ \bullet \\ \\ \end{pmatrix} \bullet \frac{\Delta t^2}{2} \Delta\ddot{\vec{r}} \qquad \text{(S4.7)}$$

The coefficient c_i depends on the order of algorithm used and also on the type differential equation used. In the current simulation algorithm of fifth order is used for which the values of c_i are as given below-

C_0 = 19/90, C_1 =3/4, C_2=1, C_3 =1/2, C_4 =1/12 (S4.8)

The data is extracted and stored periodically at the interval of some predefined time steps. Simulation is terminated after a predefined time steps in all cases. All the important particle parameters used for this simulation are listed in table S4.1.

Table S4.1: Particle Properties

Number of particles	N : 10000
Particle radius	R: 0.01 m
Young's modulus	Y: 10^9 Pa
Coefficient of friction	μ: 0.5
Damping constant	A : 0.01 sec
Tangential damping constant	γ^t : 10 Nsec/m
Material density	ρ_m: 8g/cm^3
Integration time step	Δt : 10^{-6} sec
Initial density of system	ρ_s : 12.56%

S5: Implications of kinetic instability and it's relation to grain boundaries in metal

Previous work[5] has already indicated close resemblance of the structure of 3-D centripetal packing and atomic structure of supercooled monoatomic metallic liquid. It can therefore be speculated that probably the origin of grain boundaries at least for simple metallic system lies in the kinetic instabilities of the underlying transformation processes and presumably chaotic in nature. This statement, however, warrants further close scrutiny.

S6: Detailed analysis of final packing

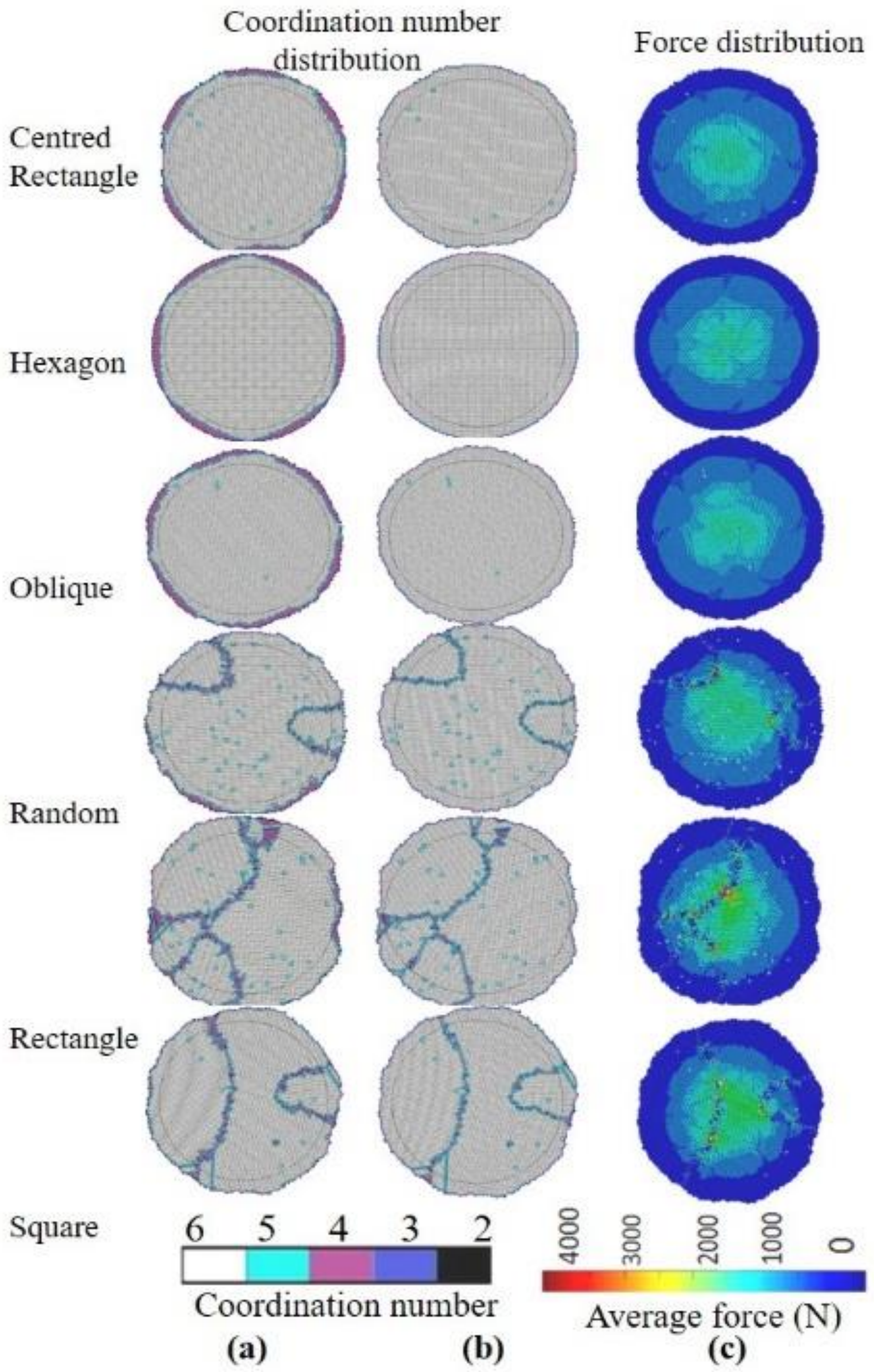

Figure S6.1: (a-b) Coordination number and (c) force distribution in final packed structure for one set of simulation for all types. Two particles are defined to be coordinated if the distance between their centres are less than *2r* and *2.1r* for (a) and (b) respectively. Because these two definitions produce identical features, for all subsequent studies the former definition is used. The force distribution plots show identical feature as coordination number distribution plots.

To eliminate the boundary effects, all the subsequent results are calculated within the circular domains that are depicted by dotted circles in Fig. S6.1a,b.

Figure S6.2 shows the pair distribution function [g(r)=<$\rho(r)$>,where, $\rho(r)=n/2\pi r dr$, and n is the number of particle centres between r and $r+dr$ from a particle of interest] of the final packing for all the six systems with no perturbation. The patterns are identical for all the systems and also to compact hexagonal packing (not shown). This is supportive of the fact that final structure is predominately hexagonal.

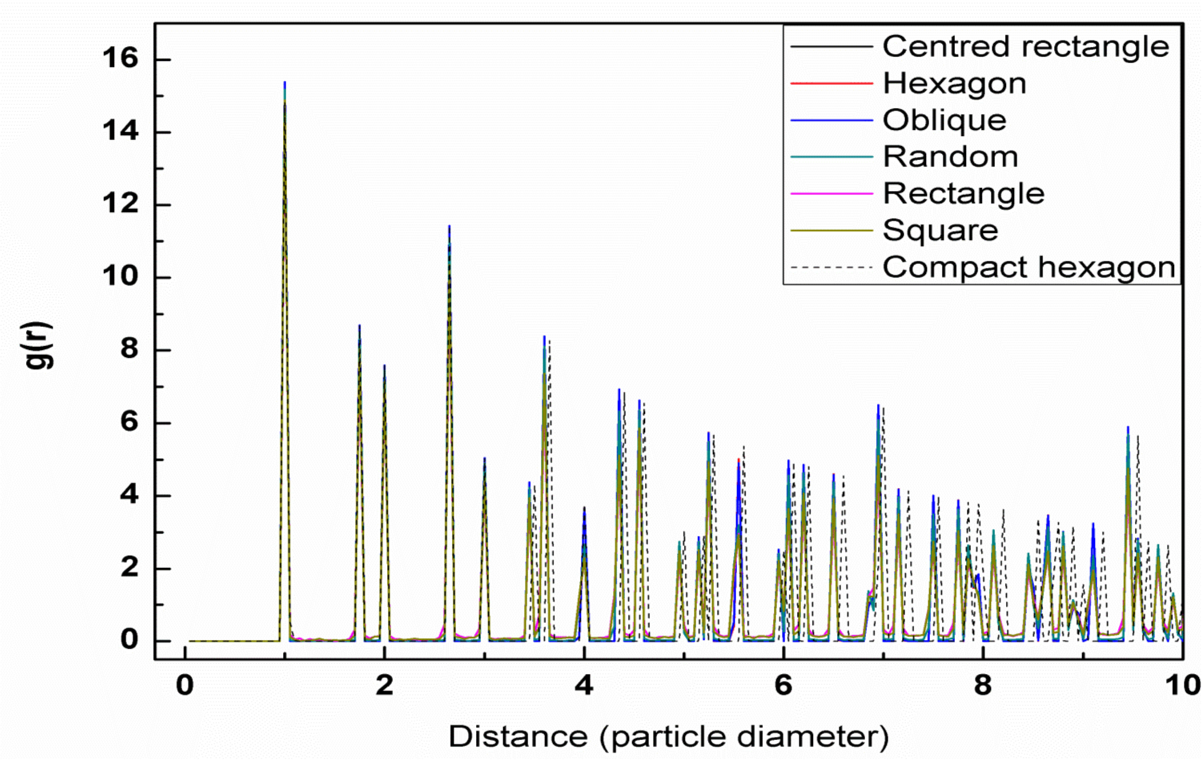

Figure S6.2: PDF of final packed structure of all six configurations with no perturbation. These patterns are nearly identical to that of the regular compact hexagonal packing supporting that the final structures are predominately hexagonal in all the cases.

Figure S6.3a shows that in final clusters, more than 85% particles have coordination number six, for five it is less than 10% and the same for two and three are insignificant (<1%). Pairwise overlap between particles are calculated for all the six types and plotted in Fig. S6.3b (systems with no perturbation). Since the values of pairwise overlap are typical for DEM simulations though the plots diverge at low values, it is not a matter of concern for the present study

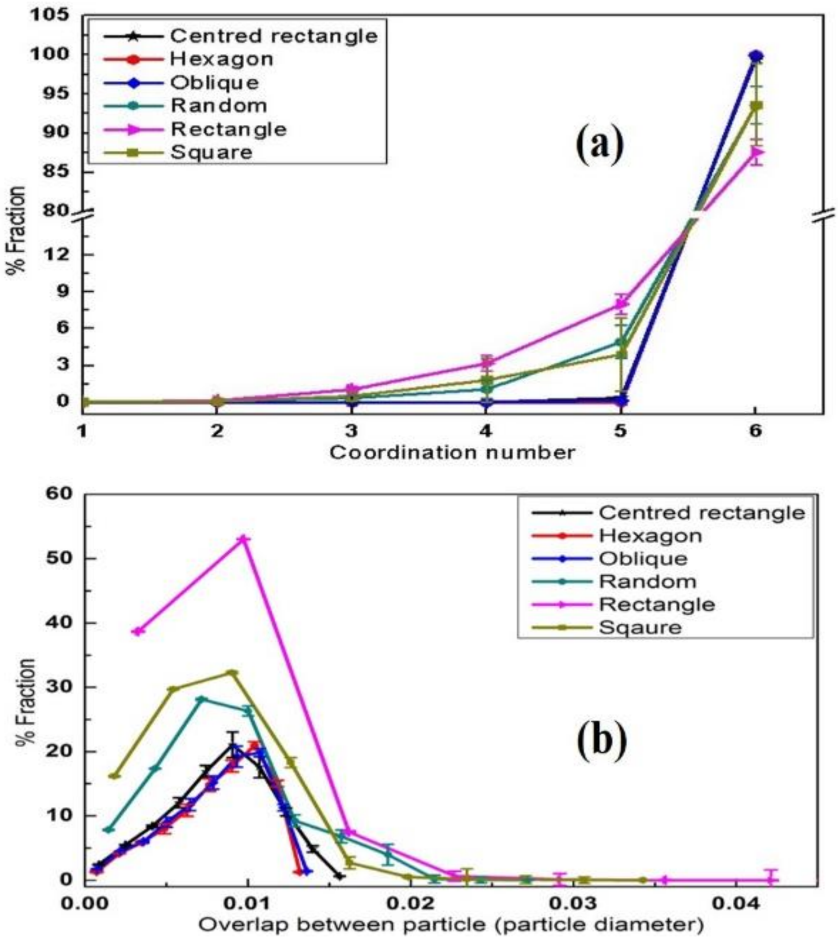

Figure S6.3: (a) Coordination number distribution of six configurations (Break plot has been used for better presentation of the result) (b) Distribution of pairwise particle overlaps

Because of the rotational invariance of the compacting force, radial distribution of coordination number is a parameter of interest and plotted in Fig. S6.4. This figure basically represents how coordination numbers of particles change as one move further from the centre of packing. It shows, in general, an inverse correlation between coordination number 5 and 6. It also shows that hexagonal, oblique, and centred rectangular (for the current choice of lattice parameters) show minimum departure from six fold coordination throughout the structure. A plausible explanation for this can be obtained from the angle distribution of Delaunay triangles shown in Fig. 1.While the angular distribution for hexagon, centred rectangle and oblique systems are narrowly distributed around the value of ($\pi/3$), the same for random, rectangle and square are more widely distributed. That is indicative of the fact that angular dispersion might be one of the parameter of prime interest and will be investigated carefully later in a quantitative way.

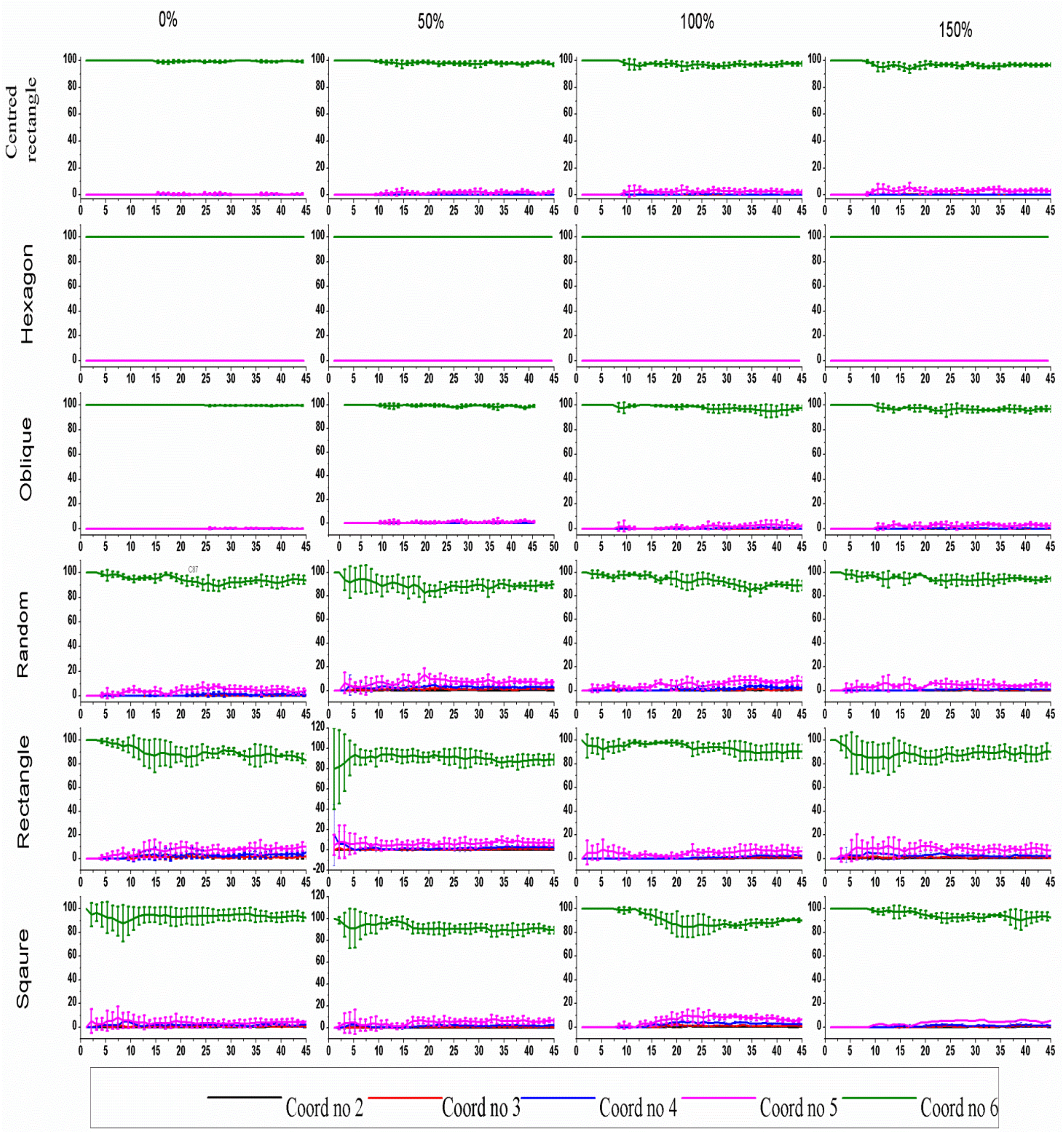

Figure S6.4: Radial distribution of coordination number of particles in final packed structure those were obtained from initial configurations of all symmetry classes with varied degrees of uniform perturbation. Figures in each row were obtained from simulation of packing using same symmetry class as initial configurations with different degrees of perturbation levels as indicating the top of each column. Y-axis represent percent fraction of particle and X-axis represent distance from centre of the final cluster where particle diameter as unit distance.

Three body distributions has been also analysed by calculating the angle distribution of Delaunay triangles with particles centre as their vertices (Fig. S6.5). The distribution shows a sharp peak at (π/3), which confirms that most of the particles in the packing have coordination number six and in agreement with other results produced until now. These data clearly indicate that irrespective of the starting configurations, all the systems finally evolve to hexagonal packing. The influence of initial symmetry in final packing is limited to the amount of disorder introduced in the predominately hexagonal packing

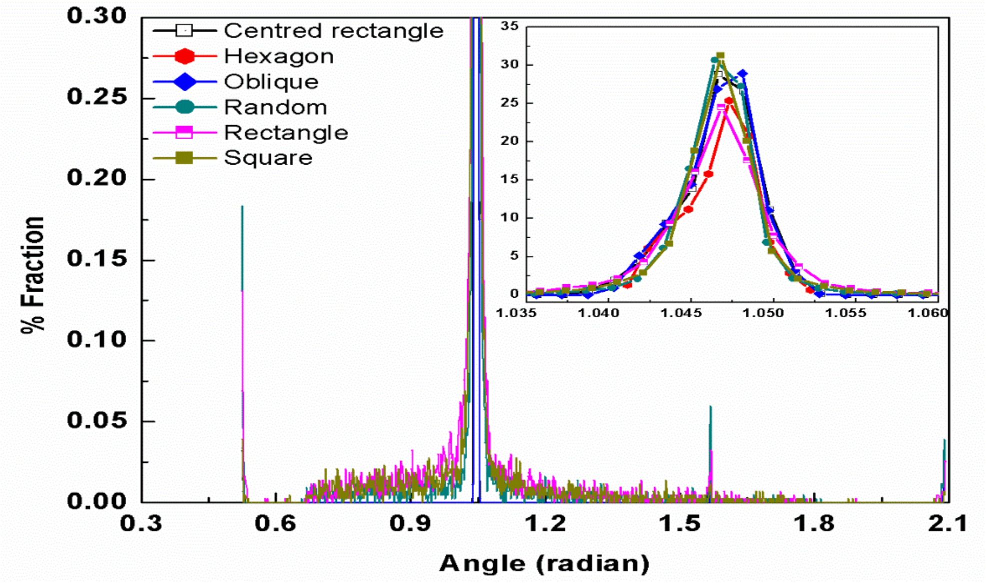

Figure S6.5: Angle distribution of Delaunay triangle

S7: Prediction of disorder in final structure

Figure S7.1a-c summarizes angular dispersion, degree of disorder and extent of disorder of final packing respectively as a function of angular dispersion of the initial configurations and three very important observations can be made:

i. Figure S7.1a shows that angular dispersion of final packing is always less than 0.1.
ii. FigureS7.1b shows that degree of disorder in final packing is always less than the angular dispersion of initial configurations.
iii. FigureS7.1c shows that extent of disorder in final packing are always less than half the angular dispersion of initial configurations.

These three statements provide predictive power for compaction of 2-d mono-sized spheres under the influence of long range force for systems with low initial densities. It is also interesting to note that that except for rectangle and random, both characterized by higher amount of angular dispersion in initial configurations, other crystal classes tend to form a group and outlined by boxes in corresponding colors. Among these three, probably (ii) is a

stronger statement than (iii) as the former deals with more detailed information than the later. Among (i) and (ii), the latter is preferred is it connects two seemingly different properties.

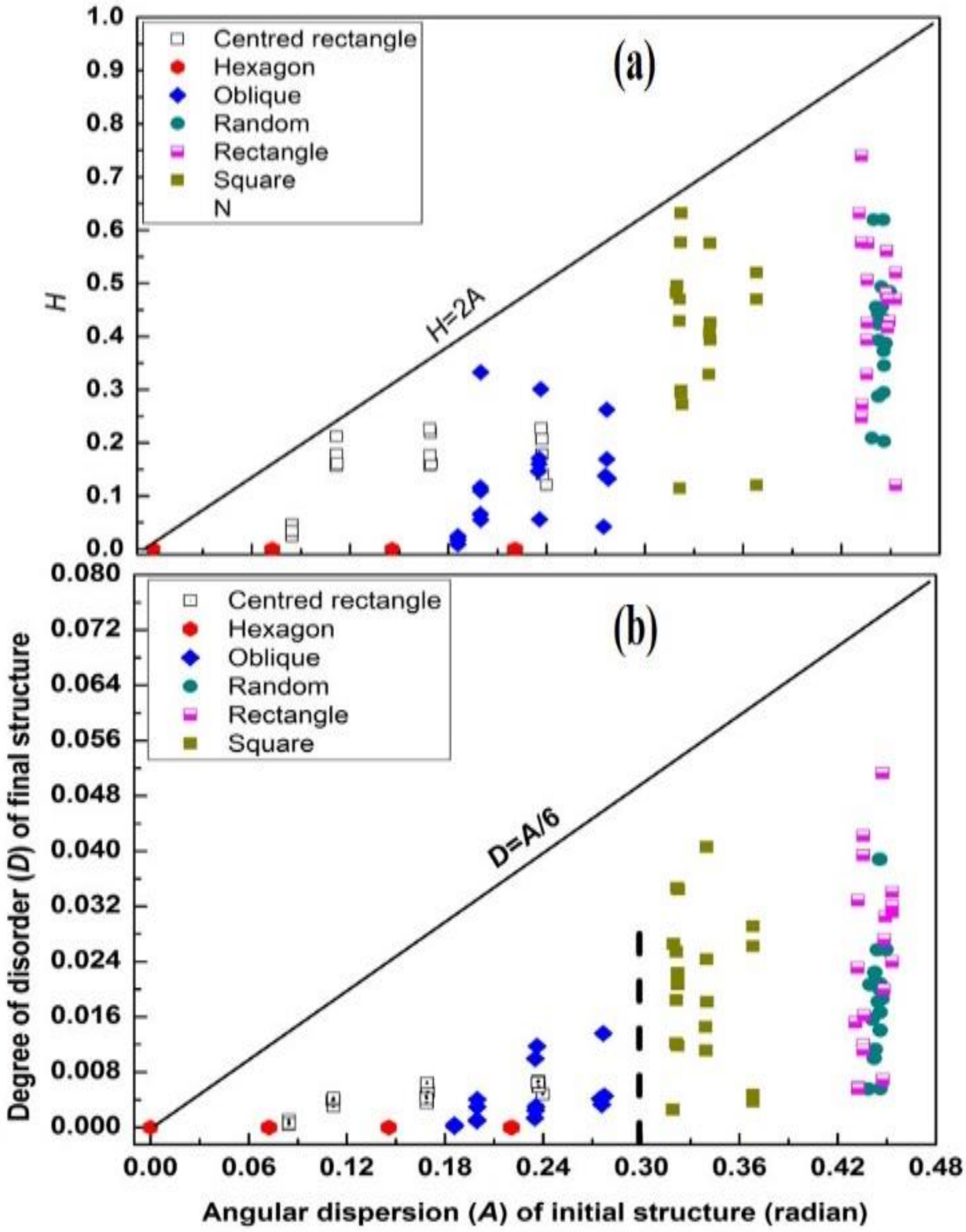

Figure S7.1: For all the simulations, (a) Entropy (H) (b) Degree of disorder (D),of final structures are plotted as a function of angular dispersion of initial configurations. Angular dispersion is measured in radians. It is observed that (a) Entropy of final structure is always less than twice of angular dispersion of initial structure (b) degree of disorder, D of final structures are always less than one sixth fraction of angular dispersion of initial configurations

REFERENCES

1 Cundall, P. A. Formulation of a three-dimensional distinct element model—Part I. A scheme to detect and represent contacts in a system composed of many polyhedral blocks. *Int. J. Rock Mech. Min. Sci. & Geomech. Abstr.* **25**, 107-116 (1988).

2 Cundall, P. A. & Strack, O. D. L. A discrete numerical model for granular assemblies. *Géotechnique* **29**, 47-65 (1979).

3 Liu, L. F., Zhang, Z. P. & Yu, A. B. Dynamic simulation of the centripetal packing of mono-sized spheres. *Physica A* **268**, 433-453 (1999).

4 Pöschel, T. & Schwager, T. *Computational Granular Dynamics*. *1st edn. Ch. 2,* 13-28 (Springer Science & Business Media, 2005).

5 Sahu, K. K., Wessels, V., Kelton, K. F. & Löffler, J. F. Testing of Frank's hypothesis on a containerless packing of macroscopic soft spheres and comparison with mono-atomic metallic liquids. *J. Alloys Compd.* **509**, S60-S63 (2011).

6 Yu, A. B. Discrete element method: An effective way for particle scale research of particulate matter. *Eng. Comput.* **21**, 205-214 (2004).

7 Hertz, H. Über die Berührung fester elastischer Körper. *Journal für die reine und angewandte Mathematik,* ***92***, 156-171 (1882).

8 Brilliantov, N. V., Spahn, F., Hertzsch, J. M. & Pöschel, T. Model for collisions in granular gases. *Phy. Rev. E* **53**, 5382-5392 (1996).

9 Haff, P. K. & Werner, B. T. Computer simulation of the mechanical sorting of grains. *Powder Technol.* **48**, 239-245 (1986).

10 Verlet, L. Computer "Experiments" on classical fluids. I. Thermodynamical properties of Lennard-Jones molecules. *Phys. Rev.* **159**, 98-103 (1967).

11 Pöschel, T. & Schwager, T. *Computational Granular Dynamics 1st edn. Ch.* 2, 54-61 (Springer Science & Business Media, 2005).
